# Progress of research on tumor organoids: A bibliometric analysis of relevant publications from 2011 to 2021

**DOI:** 10.3389/fonc.2023.1092870

**Published:** 2023-01-26

**Authors:** Yin Shuoxin, Wang Shuping, Zhang Xinyue, Zhang Tao, Chen Yuanneng

**Affiliations:** ^1^ Graduate School of Guangxi University of Chinese Medicine, Nanning, Guangxi, China; ^2^ Department of Gastroenterology, Ruikang Hospital Affiliated to Guangxi University of Chinese Medicine, Nanning, Guangxi, China

**Keywords:** tumor organoids, bibliometric analysis, drug screening, precise medicine, antioxidant polyphenols, CiteSpace, VOSviewer

## Abstract

**Background:**

Research on tumor organoids has developed rapidly over the past 20 years, but a systematic analysis of current research trends is lacking. Researchers in the field need relevant references and knowledge of current research hot spots. Bibliometric analysis and visualization is a systematic method of acquiring an in-depth understanding of the status of research on tumor organoids.

**Methods:**

CiteSpace, VOSviewer and the Bibliometric Online Analysis Platform from the Web of Science Core Collection were used to analyze and predict publishing trends and research hot spots worldwide in the field of tumor organoids.

**Results:**

A total of 3,666 publications on tumor organoids were retrieved, and 2,939 eligible articles were included in the final analysis. The number of publications has grown significantly, with the United States of America as the leading country for research on tumor organoids. Among journals, *Cancers* published the largest number of articles. Harvard Medical School published the highest number of articles among all institutions. The Chinese Academy of Sciences was ranked highest among all contributing institutions on the importance of their publications. A trend in multi-disciplinary collaboration was observed in studies on tumor organoids. Keywords indicated that the current research largely concentrated on optimizing the construction of organoid models to use for medication development and screening in the clinical setting, and to provide patients with individualized treatment for gastric cancer and colorectal cancer, which are newly emerging research hotspots. Gastric and colorectal cancers were the top two tumors that have received increasing attention and have become the focal points of recent studies.

**Conclusion:**

This study analyzed 2,939 publications covering the topic of tumor organoids. Although optimizing the construction of organoid models has always been a hot topic in this field, the application of tumor organoids to the development of medications and screenings will foster individualized treatment for patients, which is another emerging hot spot in this field of research.

## Introduction

The International Agency for Research on Cancer reported 19.3 million new cases of cancer and approximately 10 million deaths due to cancer worldwide in 2020. Approximately 28.4 million new cases of cancer are expected worldwide in 2040, which is an increase of 47%. Cancer has become a key problem endangering global public health (1). In recent years, the treatment of patients with cancer has evolved from interventions based on tumor types to those based on the tumor’s molecular characteristics or microenvironment. This approach, known as precision medicine or individualized treatment, has saved many patients with advanced cancer. At present, commonly used tumor models, such as the 2D cell culture (2), genetically engineered mouse models (3) and the human-derived tumor xenograft models (4), although essential for tumor research, are difficult to simulate perfectly the actual state of tumors *in vivo*, resulting in the failures of many clinical trials. Organoids are derived from the stem cells of 3D cultures, which can reproduce the structural and functional characteristics of a native organ and simulate the development of diseases of human organs in culture dishes (5). Tumor organoids are *in vitro* models established by surgical resection or tissue biopsy to obtain tumor tissue in patients, followed by mincing and enzymatic hydrolysis of tumor tissue and the 3D culture of the tumor cells in it (6). Compared with traditional tumor models, tumor organoids not only reflect the genetic characteristics and tissue structure heterogeneity of a patient’s tumor tissues, but they also maintain gene stability during self-renewal and throughout long-term expansion. Given these reasons, they can be used to study cancers caused by infection or gene mutation, and have unique advantages for clinical drug development and guiding individualized treatment (7).

Although the research on tumor organoids has developed rapidly in recent years, a scientific and systematic analysis of their status and trends is lacking. Therefore, we used the Web of Science Core Collection (WoSCC) as our data source to perform a quantitative analysis of the literature in the field of tumor organoid research, in order to understand and determine the research hot spots and frontiers, and to provide a relevant reference for scientific researchers in the field.

## Materials and methods

### Data sources and search strategies

**Figure 1:**
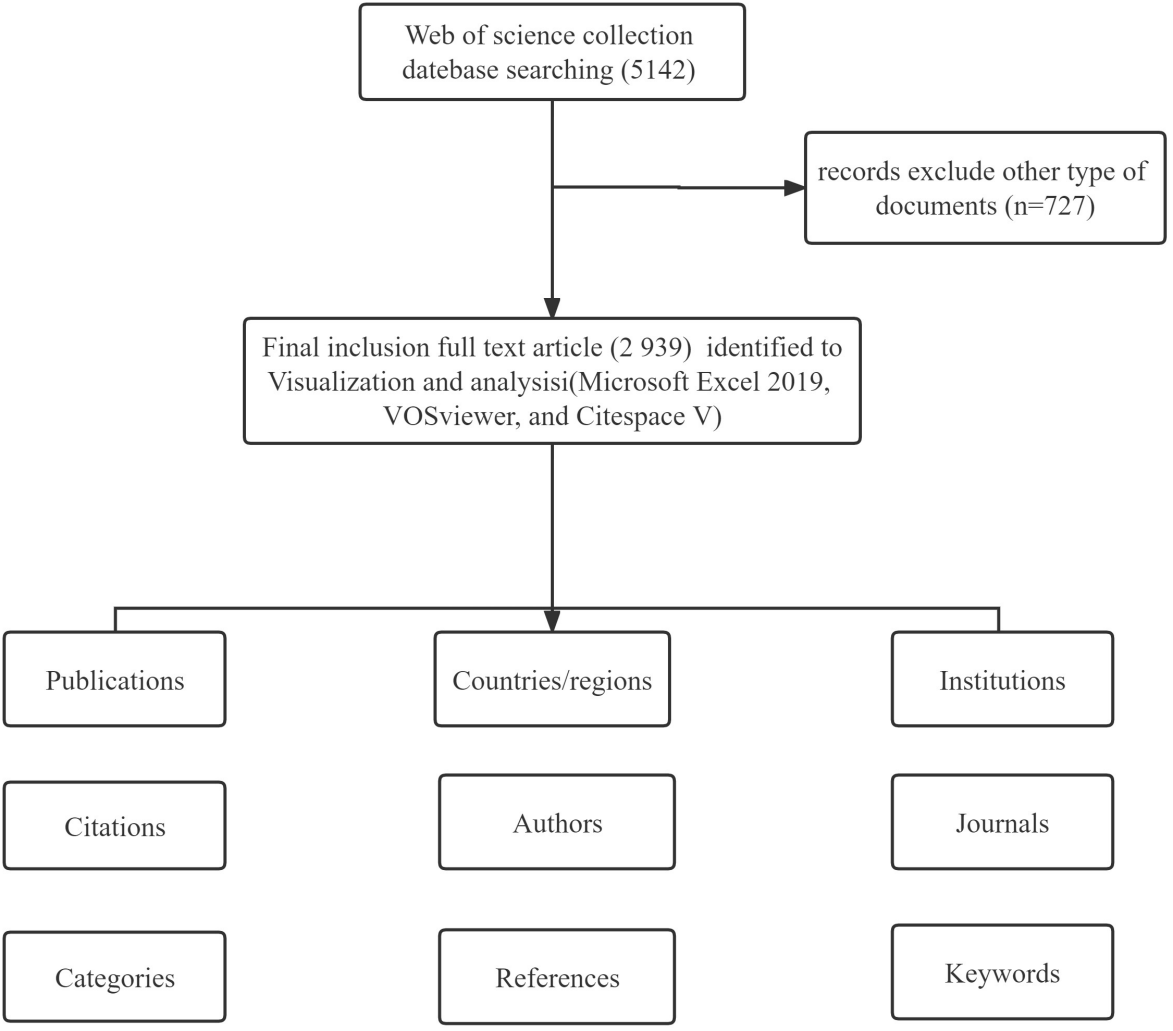
Flowchart of the research literature selection.

All of the data in this study were derived from the WoSCC. The search terms were: Tumor or Neoplasm (TS) = (“Cancer” or “Tumor” or “Neoplasm” or “Neoplasia” or “Malignant Neoplasm” or “Malignancy” or “Malignant Neoplasms” or “Benign Neoplasms” or “carcinoma”) AND (“Organoid” or “Organoids”) AND Language = English. The search period was from January 1, 2011 to December 31, 2021 and the required language was English. A total of 3,666 articles were retrieved and 727 were excluded from the analysis because either they were not research reports or they were review articles. Thus, 2,939 articles were included in the final sample for the subsequent metrological analysis, as shown in **Figure 1**. All data in the text were extracted on September 14, 2022 (the same day) to avoid deviations due to daily updates of the database.

### Study procedures

The full records and cited references of the eligible publications were downloaded from the WoSCC database and saved in TXT format; then they were imported into the CiteSpace software V5.8.R3 SE, 64 bits. CiteSpace is visual analysis software based on the JAVA environment developed by Chen Chaomei. The software is designed to analyze information in the scientific literature. The user can detect the development of rules and the distribution of scientific knowledge in a field through visualization, and identify key points in the development of a field of study based on the existing data, especially turning points and key points in the field of knowledge (8). VOSviewer is visualization software developed by the Science and Technology Research Center in Leiden, the Netherlands. The user can also build a visual network map based on information in the scientific literature and acquire a comprehensive understanding of trends in the scientific structure and dynamic development of a field (9). The full records and cited references of these publications were downloaded from the WoSCC database, saved as a tab-delimited file and imported into the Bibliometrics Online Analysis Platform^1^. We chose the “total literature analysis” option to examine trends in publications of different countries and the “partnership analysis” option to explore collaborations between countries and regions.

### Study metrics

In this bibliometric analysis, the quantity and quality of the research results were evaluated using indicators, such as the number of articles published, the frequency of citations of the article and the impact factor, which reflect the level of research and academic status of a region, institution or author in a certain field. The number of papers published is an important indicator for evaluating the capacity for scientific research output. Frequency of citations in bibliometric analysis, refers to the number of times a published paper is cited in other articles, which reflect the value of the paper to a field and the degree of attention it has received. The H-index refers to rankings of articles published using relevant statistical measures, based on an articles’ frequency of citations (from high to low). Articles with the lowest H-index have the least amount of citations, which we used as a measure to assess the number of scientific research outputs and the quality of the research in this bibliometric analysis. The journal’s impact factor and other metrics were retrieved from the 2021 edition of Journal Citation Reports. These are important indicators of the academic level and quality of the journals and the articles.

## Results

### Changing trend in the quantity of articles published

**Figure 2:**
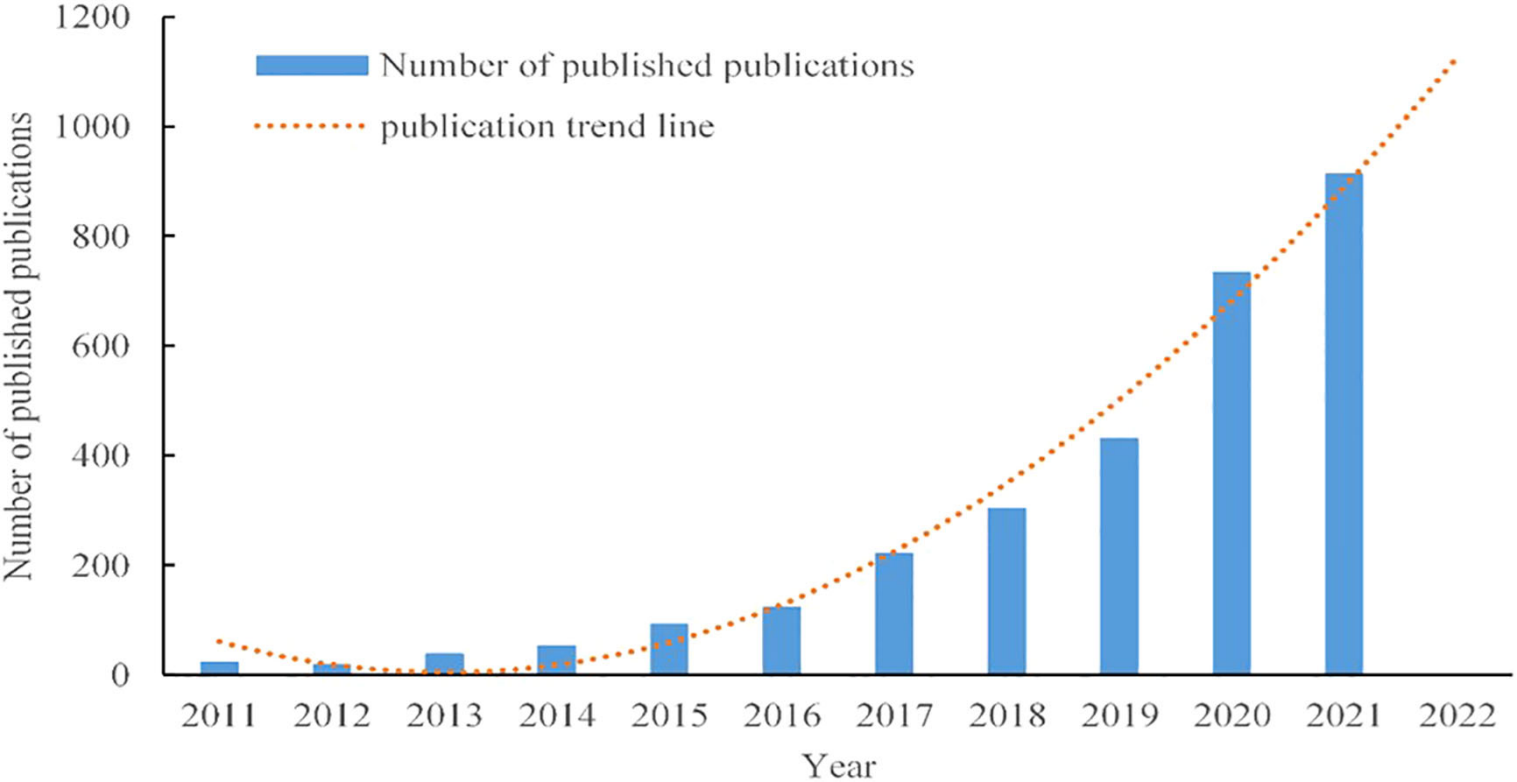
Upward trends in the volume of published research on tumor organoids from 2011 to 2021.

To clarify the rate of the development of research on tumor organoids, global trends in the volume of published research articles from 2011 to 2021 were plotted, as shown in **Figure 2**. Only 129 articles were published worldwide from 2011 to 2014. With the global shift to a rapid growth of research on tumor organoids before and after 2017, as of 2021, 912 studies have been published worldwide, accounting for 31.03% of the total number of articles published from 2011 to 2021, indicating that research on tumor organoids has become a focus of global enquiry. Given the upward trend line in **Figure 2**, the global number of publications in 2022 is expected to be significantly higher than the number of articles published last year.

### Cooperation between research publishing countries and institutions

**Figure 3:**
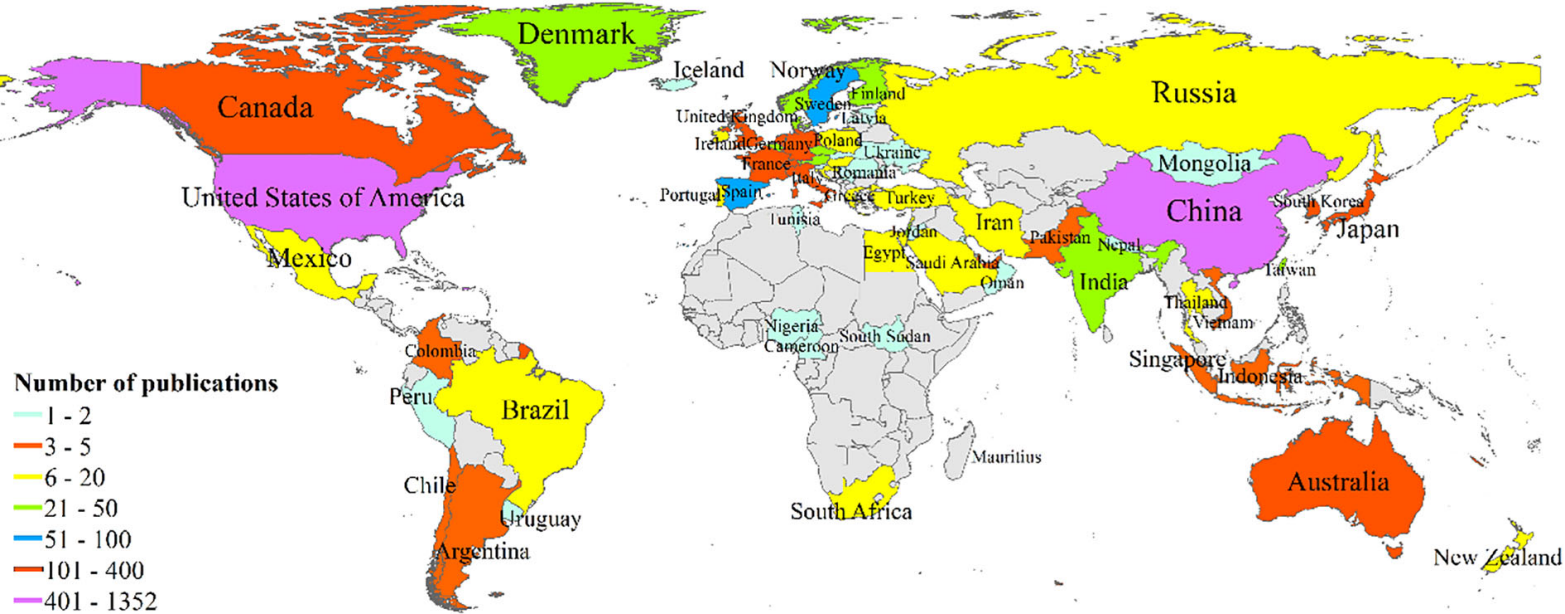
Distribution of publications by country and region based on frequencies.

Between 2011 and 2021, there were 72 countries worldwide that published research on tumor organoids, as shown in **Figure 3**. Indicators of the top ten countries with the highest number of articles are shown in **Table 1**. The highest and most significant number of articles were published in the United States of America (USA) (1,320, 44.91%) and China (447, 15.21%), followed by Germany (329, 11.19%), Japan (297, 10.10%) and the Netherlands (289, 9.83%). The H-indices of the USA, the Netherlands and Germany were 93, 67 and 48, respectively, ranking them among the top three countries with the highest H-indices. Articles from the USA, the Netherlands and England with 44,814, 25,368 and 10,089 citations, respectively, were ranked among the top three countries with the highest total number of citations, and articles from the Netherlands, England and Switzerland, with an average of 87.78, 42.93 and 36.17 citations per paper respectively, were ranked among the top three countries for the highest average number of citations per article.

**Table 1:** Publication metrics of articles on tumor organoids of the top ten countries by number of publications.

Rank	Country	Number of publications	Total frequency of citations	Average number of citations per paper	H-index
1	USA	1 320	44 814	33.95	94
2	China	477	7 525	15.78	43
3	Germany	329	8 314	25.27	48
4	Japan	297	9794	32.98	43
5	Netherlands	289	25 368	87.78	67
6	England	235	10 089	42.93	45
7	Italy	164	4 619	28.16	28
8	Canada	141	4 071	28.87	31
9	France	121	2 243	18.54	22
10	Switzerland	115	4 160	36.17	25

USA, United States of America.

**Figure 4:**
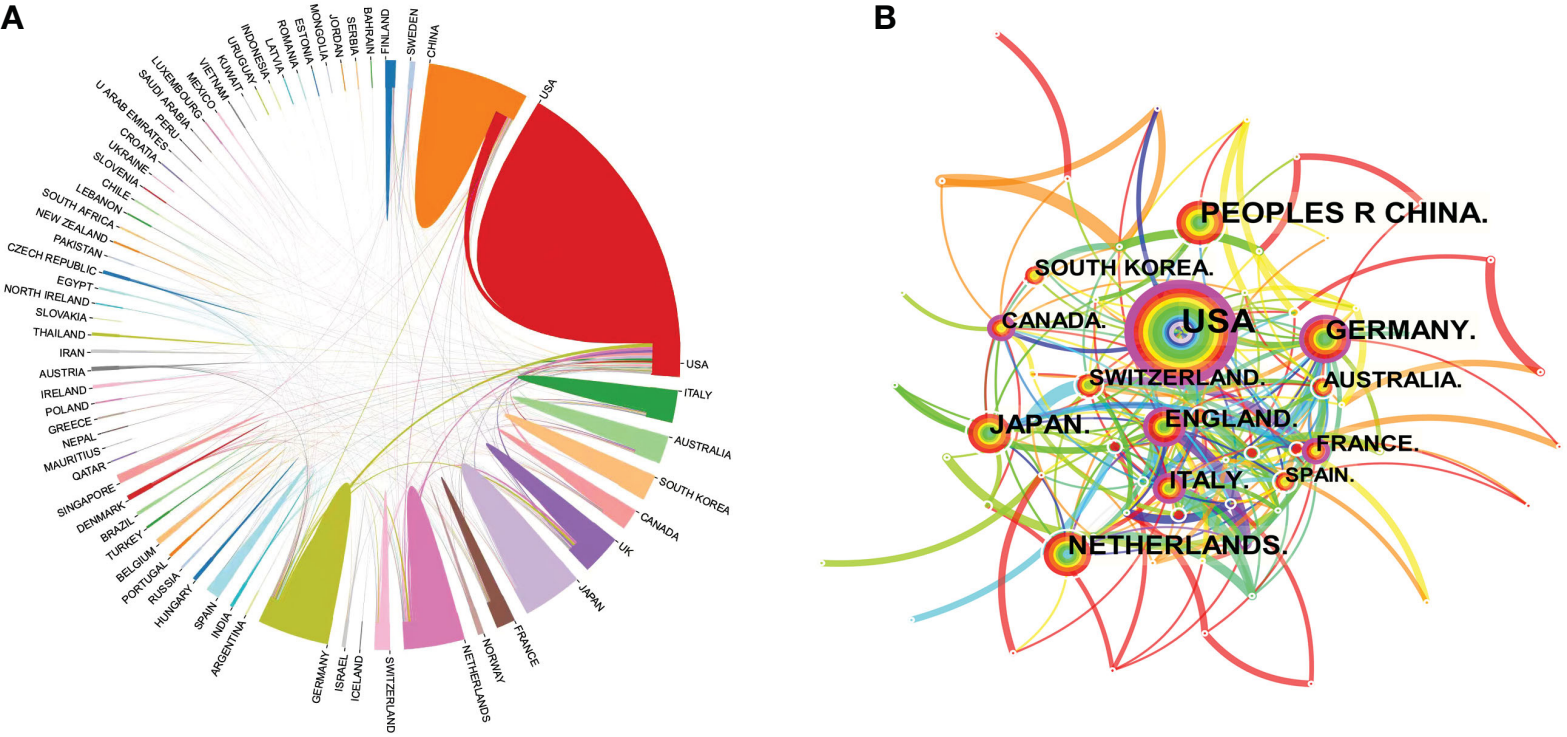
Map of the co-occurrence of tumor organoid research by nation. Figure 4 **(A)** illustrates the international cooperation among countries. These countries are represented by different colors; the links represent international cooperation and links between countries represent connections between countries. Figure 4 **(B)** shows the centrality of the articles. Each node represents a country, and the node size represents the number of articles published by the country. The larger the node, the more articles that were published. The purple color in the outermost ring represents a central node or the central location of the cooperation between the institution and other institutions.

All relevant information was exported from the WoSCC database and saved as a tab-delimited file. International cooperation between the countries publishing relevant research was analyzed using the Bibliometric Online Analysis Platform^1^, as shown in **Figure 4A**, with the USA having the highest frequency of international cooperation, followed by Germany and the Netherlands. China had close cooperative relationships with the USA, the Netherlands and Singapore. The mediation centrality of the publishing countries was analyzed using CiteSpace software, as shown in **Figure 4B**. Mediation centrality refers to the ratio of the shortest path passing through a certain point and connecting these two points in the network to the total number of shortest path lines between these two points, which is an indicator used to characterize the importance of the node. The numerical value indicates that the node is in a key position in the network and has influence. The information in **Figure 4B** was exported to report the rankings of centrality of the top five nations’ publications in **Table 2**. The USA (0.38), Germany (0.21), the England (0.21), France (0.19) and Italy (0.19) exhibited high degrees of centrality, which is represented by purple in **Figure 4B**. The above results indicate that these countries play an important role in tumor organoid research, and the academic influence of their research results influence countries worldwide. The reliability, quality and innovation of the relevant research from these countries are far ahead of other countries.

**Table 2:** Centrality rankings of the top five nations’ publications in the field of tumor organoids.

Country	Year	Centrality	Number of publications
USA	2011	0.33	1 320
Germany	2013	0.15	329
England	2011	0.13	235
France	2011	0.12	121
Italy	2012	0.11	164

USA, United States of America.

**Figure 5:**
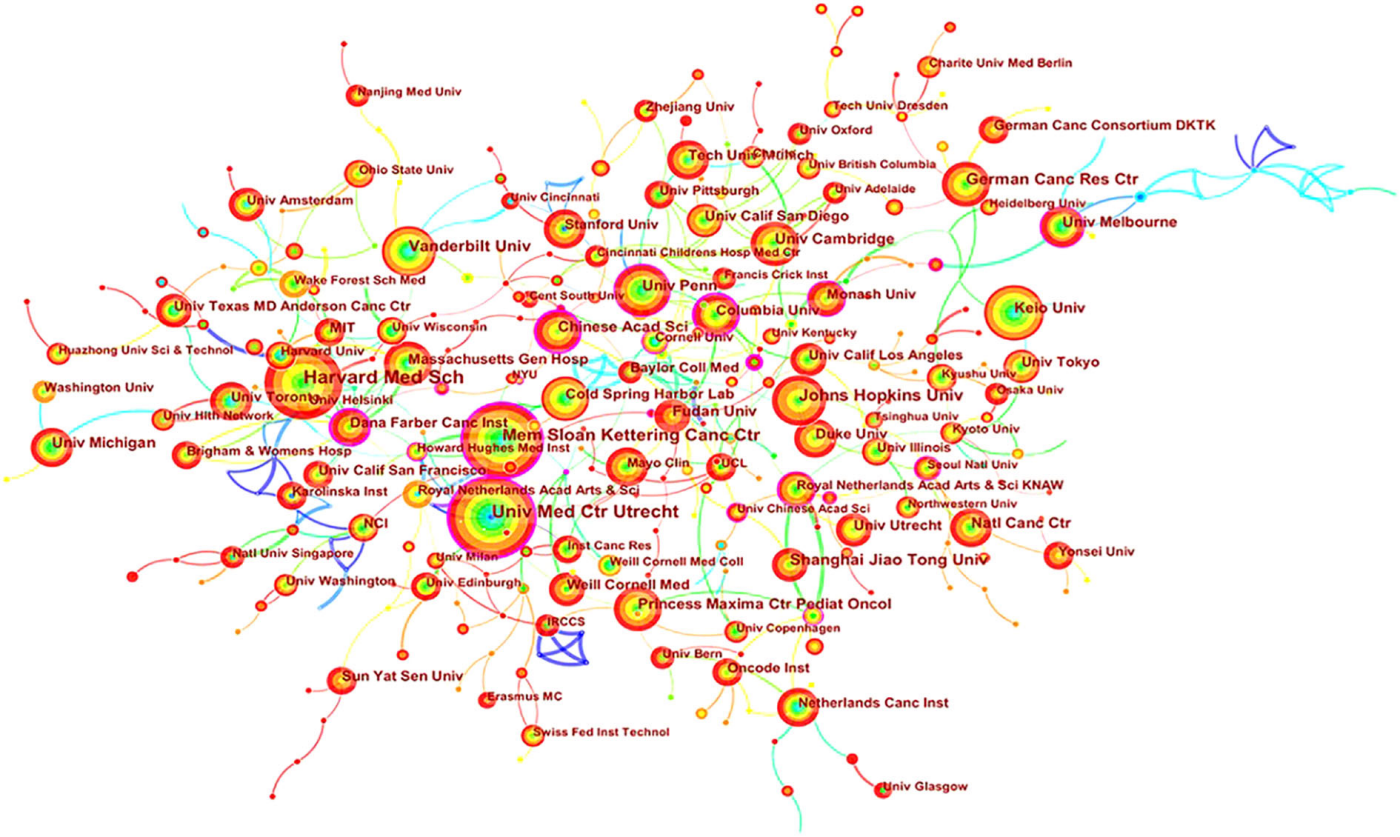
Map of cooperation in tumor organoid research. The nodes in Figure 5 represent different institutions, and the size of the nodes represents the number of articles published. The purple ring in the outermost circle indicates a central node, which has a central position in the cooperation between this institution and other institutions. The wired lines indicate cooperation among institutions.

Academic cooperation between institutions is crucial for strengthening exchanges between scholars and for disseminating advanced experiences. CiteSpace software was used in this study to conduct network co-occurrence analysis of the publishing institution, as shown in **Figure 5**. The information in **Figure 5** was exported to report the rankings of centrality and the number of publications among the top five institutions in **Table 3**. Harvard Medical School (111), the University Medical Center of Utrecht (102), Memorial Sloan Kettering Cancer Center (88), Johns Hopkins University (61) and Vanderbilt University (54) were the top five research institutions in terms of their numbers of published articles. The Chinese Academy of Sciences (0.32), Dana Farber Cancer Institute (0.30), the University of Nebraska Medical Center (0.24), Columbia University (0.23) and Memorial Sloan Kettering Cancer Center (0.22) were the top five research institutions in terms of intermediary centrality. These institutions occupy core positions in the field of tumor organoid research and engage in cooperative academic research with most institutions.

**Table 3:** Centrality rankings and number of publications of the top five institutions in the field of tumor organoid research.

Rank	Institution	Number of publications	Rank	Institution	Centrality
1	Harvard Medical School	111	1	Chinese Academy of Sciences	0.32
2	University Medical Center Utrecht	102	2	Dana Farber Cancer Institute	0.3
3	Memorial Sloan Kettering Cancer Center	88	3	University of Nebraska Medical Center	0.24
4	Johns Hopkins University	61	4	Columbia University	0.23
5	Vanderbilt University	54	5	Memorial Sloan Kettering Cancer Center	0.22

### Authors of publications and their cooperative relationships

A total of 20,139 scholars published articles in the field of tumor organoid research worldwide. The indicators of the top ten scholars as shown in **Table 4**. Four articles were published by the top ten scholars; five scholars were from the USA and three were from the Netherlands, Japan and England. Clevers H (103) at the University Medical Center of Utrecht, Sato T (34) at the Keio University School of Medicine and Chen Y (32) at Memorial Sloan Kettering Cancer Center were identified as the top three authors with the highest number of published articles. Clevers H also ranked first in the total number of citations received, the average frequency of citations per paper and the H-index. These authors have influenced tumor organoid research worldwide.

**Table 4:** Productivity, citations and H-indices of the top ten authors of tumor organoid research.

Author	Country	Number of publications	Total citations	Average number of citations per paper	H-index
Clevers H	Netherlands	103	20 939	201.34	59
Sato T	Japan	34	6 615	194.56	18
Chen Y	USA	32	2 806	87.69	19
Cuppen E	Netherlands	22	4 425	201.14	17
Van Boxtel R	Netherlands	20	4 009	200.45	15
Ewald AJ	USA	19	752	39.58	10
Tuveson DA	USA	19	3 276	172.42	17
Skardal A	USA	18	899	49.94	14
Sansom OJ	England	18	1 008	56.00	9
Sawyers CL	USA	14	2 000	117.65	14

USA, United States of America.

**Figure 6:**
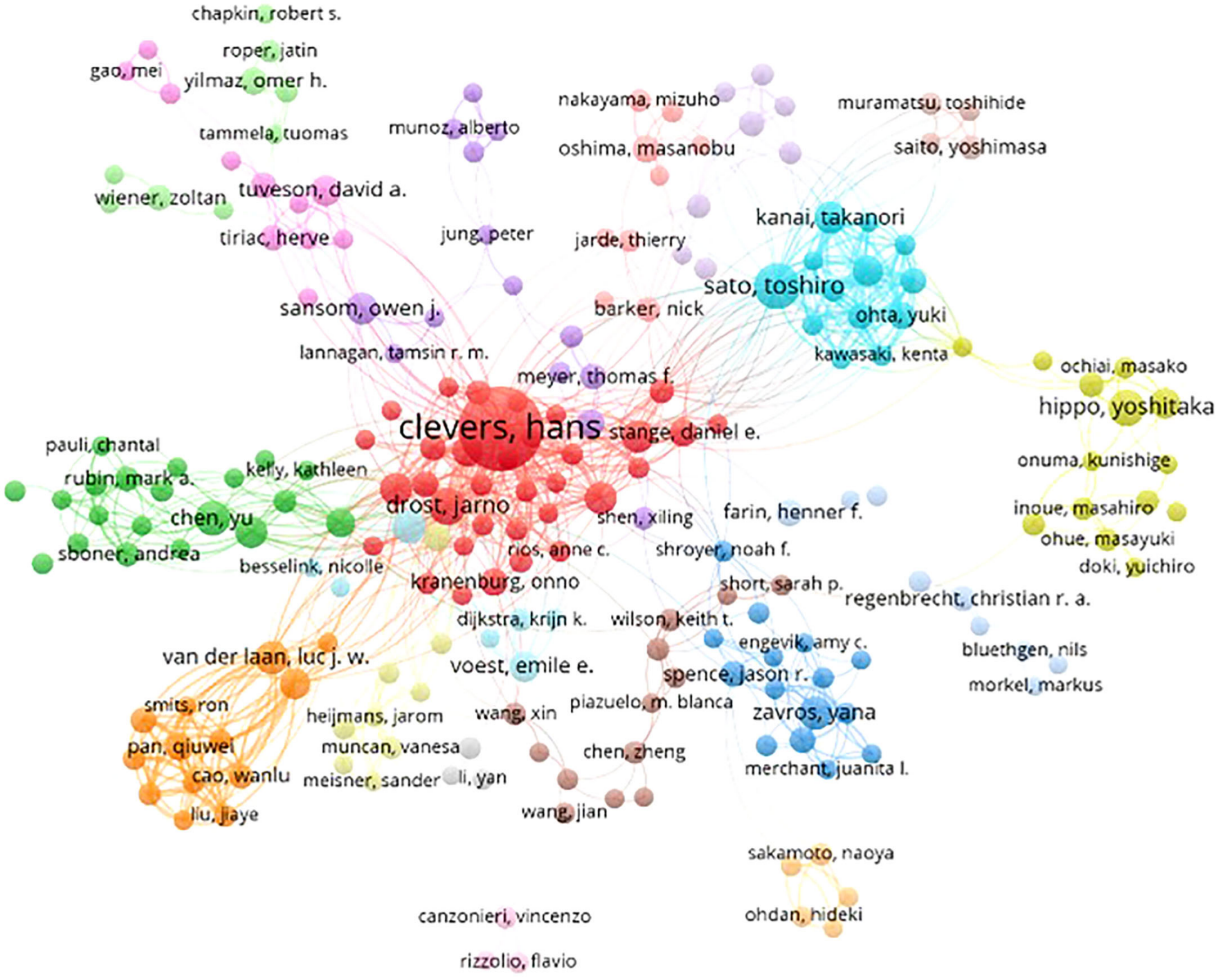
Collaborative networks among authors with a high publication volume of tumor organoid research.

Based on the cooperative network analysis of the authors with high numbers of published articles using the VOSviewer, we further defined “core authors” as those who had at least 5 publications, which included 316 authors in the analysis. We found some collaborative research teams had a high publication volume, as shown in **Figure 6**. Clevers H, Sato T, Drost J, Chen Y, Sansom OJ, Braker N and Van Der collaborated closely, but most of the inter-author collaborations were limited to intra-team collaborations, with little international collaboration, such as several small cooperative networks in the periphery (e.g., Hippo Y, Onuma K and Inoue M., and Jun P, Meyer TF and Bartfeld S). If cooperative research between authors can be strengthened, especially between authors from different countries or institutions, exchanges and innovations will likely improve significantly.

### Journal publications of tumor organoid research

Journals play a crucial role in promoting international cooperation and progress in the level of scientific research. Between 2011 and 2021, 752 journals worldwide published academic papers on tumor organoid research, of which the top ten journals published 5 papers on the topic as shown in **Table 5**. *Cancers* published the most research results, with 130 articles, accounting for 4.42% of the total publication volume, followed by *Scientific Reports* and *Nature Communications*, which published 85 and 74 articles, respectively, accounting for 2.89% and 2.51% of the total volume of publications. *Gastroenterology* ranked first in both citation frequency and the H-index. Among the top ten journals, eight journals belonged to partition Q1, 2 belonged to partition Q2 and 1 to partition Q3, with an impact factor ranging from 1.696 to 23.937, and *Gastroenterology* having the highest impact factor. The analysis shows that these international journals were the academic authorities on the findings of tumor organoid research, which were most likely to be of concern to scholars in various countries.

**Table 5:** The top ten journals that published papers on tumor organoid research.

Rank	Journal	Count	Average number of citations per paper	H-index	Impact factors	Zone
1	*Cancers*	130	6.55	16	6.999	Q1
2	*Scientific Reports*	85	17.21	19	5.134	Q1
3	*Nature Communications*	74	30.57	26	15.805	Q1
4	*Gastroenterology*	54	81.30	27	23.937	Q1
5	*Cancer Research*	50	28.14	19	12.843	Q1
6	*Oncogene*	48	17.71	18	8.858	Q1
7	*Cell Reports*	44	35.70	24	10.394	Q1
8	*Frontiers in Cell and Developmental Biology*	44	4.89	9	7.219	Q1
9	*Cells*	43	14.00	14	6.663	Q2
10	*Jove Journal of Visualized Experiments*	40	7.83	8	1.696	Q3

### Research directions

**Figure 7:**
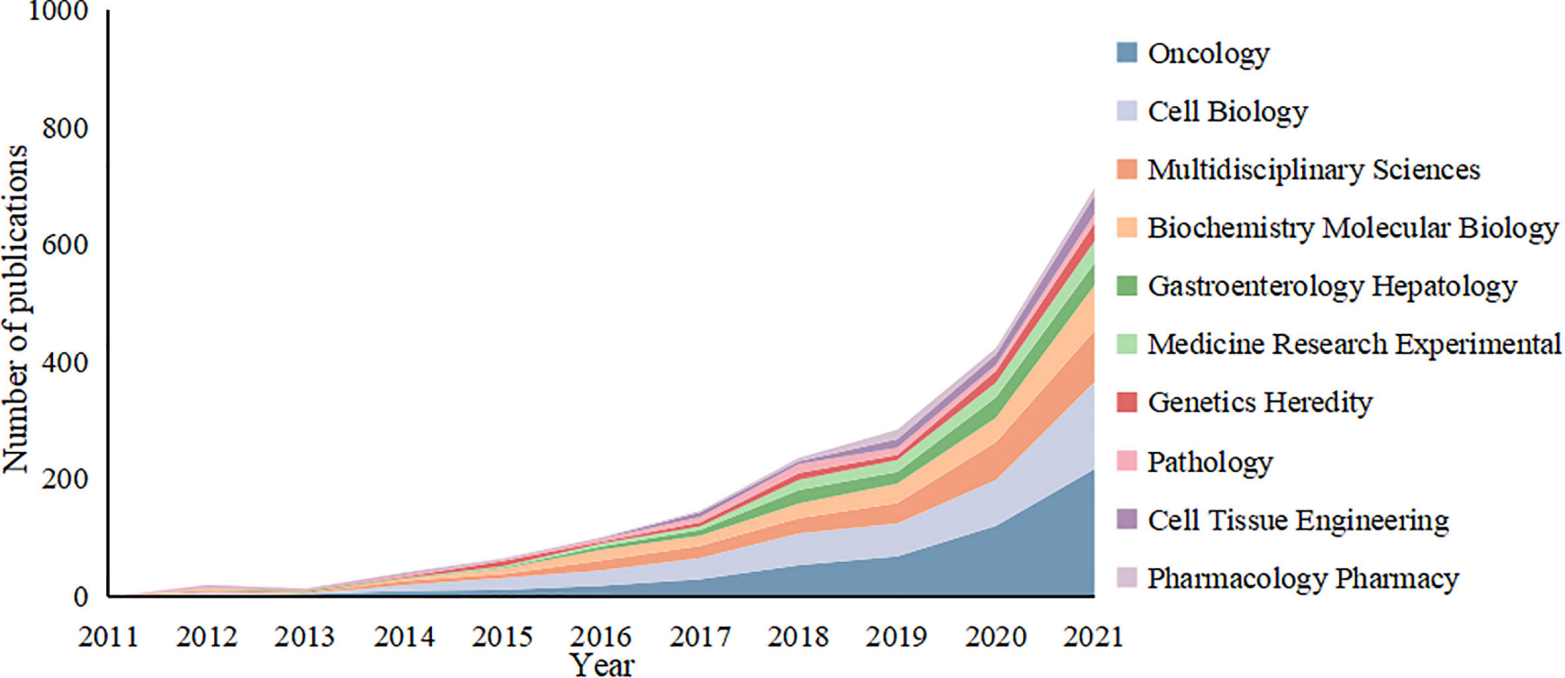
Variation curve of the number of published papers by main research direction of the tumor organoid research.

The 2,939 articles in this study were classified into 95 research directions. It should be noted that the sum of the percentages of all the studies’ directions exceeded 100% because the same article could be classified into multiple research directions. Changing trends in research directions in recent years is shown in **Figure 7**. The most articles published were in the direction of Oncology with a total of 802, accounting for 27.25% of the total number of articles published. These were followed by Cell Biology and Multidisciplinary Sciences, with 599 and 362 articles, respectively, followed by Biochemistry Molecular Biology and Gastroenterology Hepatology. Experimental Medicine Research, Genetics Heredity, Pathology, Cell Tissue Engineering and Pharmacology Pharmacy, with more than 100 articles. Thus, this study of tumor organoids showed the developing characteristics of multidisciplinary intersection. Given the changes in the number of articles published in each research direction over time, the top five research directions of the articles indicated an increasing trend in fluctuations before 2016. All research directions reached a state of rapid growth after 2016, with Oncology developing most rapidly.

### Frequently cited papers

The total frequencies of citations of the top ten articles were derived from the citation reports of the WoSCC. These articles were cited more than 600 times, of which 9 were from the team led by Clevers H (**Table 6**). In 2011, the Clevers H team successfully constructed a small intestinal crypt-villus structure *in vitro* without mesenchymal conditions from a single LGR5 stem cell (10). The team then added different growth factors or inhibitors to different organoids, based on previous studies, so that the replication potential of the adult stem cells was not limited *in vitro*. This improvement promoted the use of organoid models as a tool for investigating diseases with increasing complexity (11–13). The team also constructed a variety of tumor organoid models, such as models of colorectal, prostate and pancreatic cancers (14–19). These constructed models had a better fit with real tumor growth and development, and the organoid tumor models that were constructed using the patient’s own tumor cells facilitated a more individualized approach to the treatment of tumors. These studies have opened up a new path for acquiring a more in-depth understanding of the mechanism of tumorigenesis and development, and clinical drug development and screening.

**Table 6:** The total number of citations of the top ten journal articles related to tumor organoids.

No.	Journal articles	Citation	Year
1	Long-term Expansion of Epithelial Organoids From Human Colon, Adenoma, Adenocarcinoma, and Barrett’s Epithelium	1740	2011
2	Paneth cells constitute the niche for Lgr5 stem cells in intestinal crypts	1520	2011
3	Modeling Development and Disease with Organoids	1116	2016
4	Prospective Derivation of a Living Organoid Biobank of Colorectal Cancer Patients	1093	2015
5	Organoid Models of Human and Mouse Ductal Pancreatic Cancer	971	2015
6	Distinct populations of inflammatory fibroblasts and myofibroblasts in pancreatic cancer	793	2017
7	Organoid Cultures Derived from Patients with Advanced Prostate Cancer	768	2014
8	Growing Self-Organizing Mini-Guts from a Single Intestinal Stem Cell: Mechanism and Applications	670	2013
9	Patient-derived organoids model treatment response of metastatic gastrointestinal cancers	630	2018
10	Single-cell messenger RNA sequencing reveals rare intestinal cell types	625	2015

### Analysis of keywords

**Table 7** shows the top twenty most frequently occurring keywords analyzed by CiteSpace. Nodes were set as keywords with a time interval of 2011–2021 and yearly time slices. We found that “stem cells,” “cancer,” “*in vitro*,” “colorectal cancer,” “model,” “culture,” “differentiation” and “growth” were the most frequently occurring keywords, and “3D culture,” “tumor model,” “beta catenin,” “inflammatory bowel disease,” “small intestine,” “P53” and “cancer metabolism” were the most central keywords.

**Table 7:** The top twenty most frequently occurring keywords and their centrality in research related to tumor organoids.

No.	Keywords	Count	Centrality	No.	Keywords	Centrality	Count
1	stem cell	604	0.00	1	3d culture	0.17	69
2	Cancer	570	0.03	2	tumor model	0.17	10
3	Expression	507	0.01	3	beta catenin	0.16	72
4	*in vitro*	469	0.11	4	inflammatory bowel disease	0.12	44
5	colorectal cancer	333	0.01	5	adult stem cell	0.12	9
6	Model	292	0.03	6	*in vitro*	0.11	469
7	Cell	284	0.07	7	*in vivo*	0.11	59
8	differentiation	249	0.01	8	small intestine	0.11	44
9	Growth	196	0.05	9	p53	0.11	37
10	Culture	191	0.05	10	cancer metabolism	0.11	3

P53, Tumor suppressor protein.

A K-means cluster analysis of the keywords was performed using CiteSpace, and 19 clusters were obtained (**Table 8**; **Supplementary Figure 1**). The keyword co-occurrence network in the field of research on tumor organoids is shown in **Supplementary Figure 1**. The main research directions were divided into the following categories: #0 (cell), #1 (stem cell), #2 (generation), #3 (expression), #4 (cerebral organoid), #5 (extracellular matrix), #8 (*in vitro*), #9 (landscape), #13 (*in vitro*) and #14 (culture) the culture and characteristics of the tumor organoid models. Categories: #6 (gastric cancer), #7 (rectal cancer), #10 (inflammatory bowel disease), #12 (colorectal cancer) and #17 (cancer) were classified as diseases currently used in tumor organoids, and categories #11 (precision medicine), #15 (personalized medicine), #16 (progression) and #18 (photo thermal medicine) were classified as current research and trends in tumor organoids. These 19 clusters summarize the process of development of tumor organoid research, and highlight research hot spots.

**Table 8:** Keyword clusters from the analysis of tumor organoids.

Cluster ID	Count	Silhouette	Year	Cluster name
#0	42	0.973	2015	Cell
#1	40	0.884	2015	Stem cell
#2	39	0.882	2016	Generation
#3	39	0.882	2014	Expression
#4	36	0.848	2019	Cerebral organoid
#5	35	0.886	2017	Extracellular matrix
#6	34	0.901	2017	Gastric cancer
#7	33	0.869	2018	Rectal cancer
#8	32	0.946	2015	*In vitro* expansion
#9	31	0.938	2019	Landscape
#10	30	0.82	2017	Inflammatory bowel disease
#11	29	0.834	2018	Precision medicine
#12	27	0.908	2018	Colorectal cancer
#13	27	0.955	2016	*In vitro*
#14	25	0.924	2015	Culture
#15	22	0.879	2015	Personalized medicine
#16	21	0.873	2013	Progression
#17	21	0.942	2015	Cancer
#18	7	0.961	2018	Photothermal therapy

## Discussion

### Applications and advantages of tumor organoids

Although the 3D culture was proposed more than 100 years ago, it was not until 2009 that the first organoid model of a single stem cell source was established, and Clevers’ laboratory demonstrated that a single LGR5 stem cell could successfully construct small intestinal crypt-villous structures *in vitro* in the absence of interstitial space (20, 21). In recent years, the application of 3D culture models in cancer research has developed rapidly, and studies of almost every tumor have solved a wide variety of research problems. In contrast to the widely used 2D cell culture and animal models, organoids are an important preclinical model for cancer research with distinct advantages. The 2D cell culture is simpler, cheaper and allows more direct genetic manipulation than the 3D culture models permits; however many cell lines cannot mimic the real situation of tumors accurately due to the lack of spatial organization and the tumor’s micro-environment. Increasing the dimension of the extracellular matrix from 2D to 3D significantly improved cell proliferation, differentiation and survival, and the success rate of patient-derived tumor organoid cultures (22, 23). The patient-derived xenograft model is similar to the original tumor in terms of its histological expression and biological behavior, such as protein expression, tumor biomarker status and genetic status, which cannot be achieved by tumor organoid models (24). However, patient-derived xenograft model transplantation is difficult to perform (25) and time consuming (26), and its cost is relatively high. In addition, genetically engineered mouse models can be used to assess *in vivo* phenotypes and incorporate the complexity of the syngeneic tumor microenvironment. However, the complexities of time, cost and genetic manipulation are significant drawbacks of these models, compared to those of the organoid models (24). The advantages and disadvantages of the organoid model compared with other models are summarized in **Supplementary Figure 2**.

### Drug development and screening

Non-clinical testing was used to determine the potential risk of drugs and their efficacy in humans. However, the most commonly used 2D *in vitro* cell culture system lacked heterogeneity and could not accurately describe or mimic the rich environment *in vivo* and the complex process of disease formation. Significant flaws were also found in animal studies, with species differences causing most drugs to exhibit different drug sensitivities in different *in vitro* models (27). The 3D cell culture models have been developed to reflect drug sensitivity in humans more accurately. Compared to the 2D cell culture systems, the 3D cell culture models can replicate the complex micro-ecological environment of human organs can be used for drug safety assessments and combination studies and they can provide key pharmacokinetic and biomarker data (28–30). The intestinal epithelium is an important site for drug absorption and metabolism and plays an important role in the oral bioavailability of drugs. In particular, intestinal epithelial cells serve as gatekeepers for drug and nutrient absorption (31). The data from cell and animal models related to intestinal nutrient absorption, metabolism and oral bioavailability of drugs have poor accuracy due to tumor heterogeneity and species differences. In contrast, intestinal organoids are used to examine different aspects of bioavailability, including drug distribution, individual differences, and high-throughput screening (32). Furthermore, the establishment of organoid models for the liver, kidney and blood-brain barrier meet the basic requirements for evaluating the different pharmacological aspects of drugs (33). In addition to efficacy, drug metabolism and toxicological mechanisms are also important to assess. Simplified culture models of primary human hepatocytes or liver cell lines that are currently used differ considerably from *in vivo* physiology in terms of drug toxicity, often resulting in failed drug conversion (34). Therefore, the determination of toxicological properties relies mainly on animals. However, due to species differences between humans and animals, the results might not be consistent with the real situation (35). At present, a reproducible human liver organoid model can detect bile acid uptake and excretion in modified human liver organoids in real time by knocking out bile acid transporter genes. The assay platform can be used for large-scale compound screening and has been shown to be genotype-specific sensitive to bosentan-induced cholestasis. This powerful assay will facilitate diagnoses, functional studies, drug development and individualized therapies (36).

### Individualized treatment

Traditional cancer treatments (chemotherapy and radiation therapy) are ineffective or have a positive effect in only a subset of patients due to the heterogeneity of malignancies. To ensure the effectiveness of the treatment, it is necessary to consider the patient’s personal characteristics (37). Precision medicine has been propelled forward by current large-scale tumor sequencing efforts and numerous therapeutic targets that have been identified as a result. Despite the reports of many successful cases based on DNA sequencing, the need for effective treatment remains significant for the majority of patients with cancer (38). However, genetic changes in a small percentage of coding regions do not adequately describe the complexities of cancer progression. Although DNA sequencing provides information about cancer drivers that are relatively well-preserved during cancer progression, it does not include the effects of other regulatory factors, such as epigenetic changes or non-coding regions, which are more dynamic and their relevance is more difficult to understand (39, 40). Therefore, a dynamic and versatile model system is needed to analyze tumor biology from multiple dimensions accurately and to reflect the behavior of the original tumor in the patient. The ability of tumor organoids to retain the original tumor characteristics makes them unique in studies at the individual level of patients with cancer, and they have been proposed as a model for precision medicine. For example, a study established a bank of living tumor samples from patients with advanced rectal cancer who received adjuvant chemo-radiotherapy. The combined clinical trial data confirmed that the chemo-radiotherapy response of the patients was a very close match with the organoid response of the rectal cancer, with an accuracy of 84.43%, a sensitivity of 78.01% and a specificity of 91.97% (41). Organoids have also successfully predicted the sensitivity of colorectal cancer (42), gastric cancer (43), ovarian cancer (44) and prostate cancer (45) to drugs. It appears at the present time that extracting samples from patient tumor tissues, creating and culturing organoids, exposing patient-derived tumor organoids to various drugs to find the best drug or drug combination to treat patients, revealing potential weaknesses of individual treatments based on gene mutation profiles and treatment responses of organoids and determining the next treatment route when first-line treatment is ineffective will become the ultimate mode of treatment for cancer (46, 47)^1^.

According to the latest literature and further analysis of keywords, the chemopreventive and therapeutic effects of antioxidant polyphenols on tumors were found to have received increasing attention from the scientific community in recent years. In an examination of antioxidant polyphenols, the complex technology of organoids as a new anti-cancer strategy has displayed unique advantages. Polyphenols that largely exist in plants serve as reactive oxygen species scavengers with prominent antioxidant activity, which exert the inhibitory effects on inflammation by manipulating the inflammation-related signaling pathways and suppressing the release of inflammatory mediators (48, 49). Not only contributing to treating non-neoplastic diseases, such as natural inducers from saffron targeting Nrf2/vitagene pathway to suppress oxidative stress and neuroinflammation and consequently inhibiting cognitive dysfunction (50), polyphenols could also play their roles in preventing and treating tumors by regulating cell apoptosis, autophagy, cell-cycle progression, inflammation, invasion, and metastasis through the activation of tumor suppressor genes and the inhibition of oncogenes (51, 52). Professor Scuto M’s team demonstrated that, polyphenols with low concentrations could suppress oxidative stress and inflammation, and induced the apoptotic of brain cancer cells by activating Nrf2/vitagene pathway (53), in addition, the supplementation of dietary polyphenols and vitamin D can also manipulate oxidative stress, inflammation and autophagy dysfunction in tumor cells through Nrf2/vitagene pathway, the low concentrated polyphenols and vitamin D could serve as cytotoxic agents of pro-oxidants, in the form of molecular activation through reactive oxygen species production and several survival pathways (e. g., glutathione) to induce apoptosis and cell-cycle arrest in tumor cells, for the chemoprevention and treatment of tumors in the presence of metal ions. Furthermore, the study has fully investigated a range of potential interventions in tumor organoid models depending on plant polyphenols and vitamin D, which is required to be introduced into clinical practice combined to powerful and novel technique through future interventions incorporating redox mesums of oxidative stress (54). The establishment of tumor model aims to approach 100% simulate the practical condition of the true tumor in the patient, so as to contribute to preclinical research and clinical application. Patient-derived organoids as a stable biology could facilitate gene operation, which is suitable for high-throughput sequencing analysis, and can reproduce the development from native tissue to the whole process of tumor occurrence (55). Currently some studies have taken tumor organoids as a model to explore the effect of polyphenols in tumor treatment, the results indicated that the low-dose polyphenols combined with chemotherapeutic drugs could not only optimize the efficacy, but also alleviate cytotoxicity and prevent the drug resistance (56). Another study with patient-derived organoids model revealed that the oligomeric proanthocyanidins combined to the curcumin attenuated the expression of cyclin D1, PCNA, and HSPA 5, which could serve as a method that preferentially targets cancer cells without affecting normal cells (57). The latest development of organoid models provides an excellent preclinical platform for the research of tumors, as well as makes the personalization of tumor therapy, evaluation of drug efficacy easier, contributing to the more in-depth exploration the mechanisms of cancer.

In summary, organoids mimic the structural and functional properties of native tumors, and compared to traditional tumor models, tumor organoids maintain gene stability while self-renewing and expanding over the long-term, and reflecting the genetic characteristics and tissue structure heterogeneity of the patients’ tumor tissues. In recent years, the study of tumor organoids has steadily increased, and effective organoid cultures have made it possible to screen individualized drugs within the time line of clinical treatment and apply them to translational medicine and individualized treatment. The use of organoids as precise and high-throughput preclinical tools for precision medicine is an unavoidable future trend. CiteSpace and VOSview software were used in this paper to analyze the bibliometrics and visualize the data derived from tumor organoid research.

## Data availability statement

The original contributions presented in the study are included in the article/supplementary material. Further inquiries can be directed to the corresponding authors.

## Author contributions

YS is responsible for conception design, article writing and proofreading. WS and ZX are responsible for literature collection, analysis and summary. CY and ZT are responsible for project guidance. All authors contributed to the article and approved the submitted version.

## Footnotes

1
https://bibliometric.com
