# Supplementary figures and images for: Progress of research on tumor organoids: A bibliometric analysis of relevant publications from 2011 to 2021
Source: Front Oncol. 2023 Jan 26;13:1092870. doi: 10.3389/fonc.2023.1092870 (PMC9909405; doi:10.3389/fonc.2023.1092870)

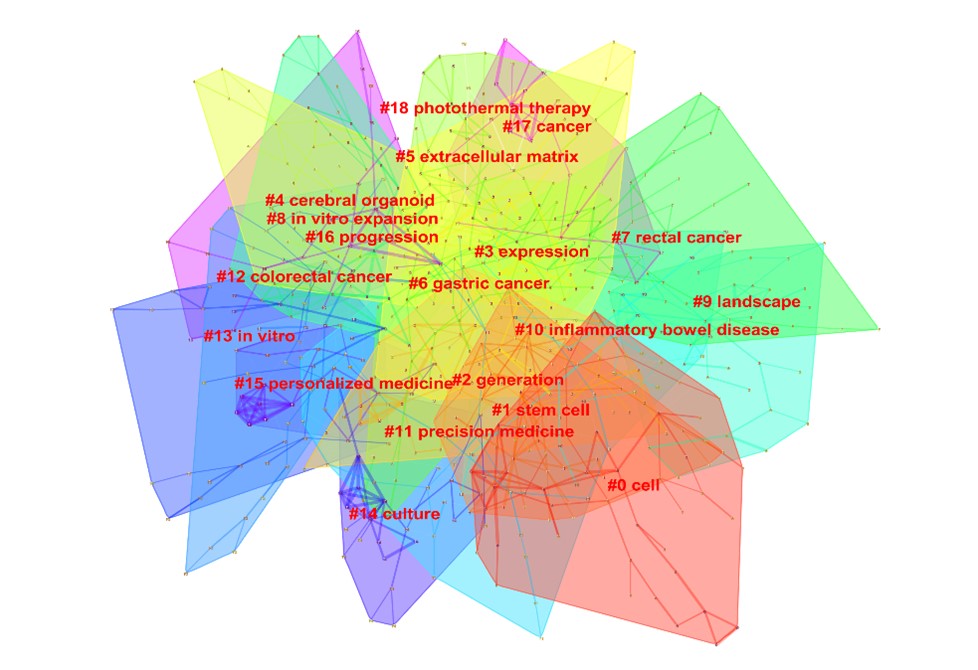

Supplement: Supplementary file 1 [file Image_1.jpeg]

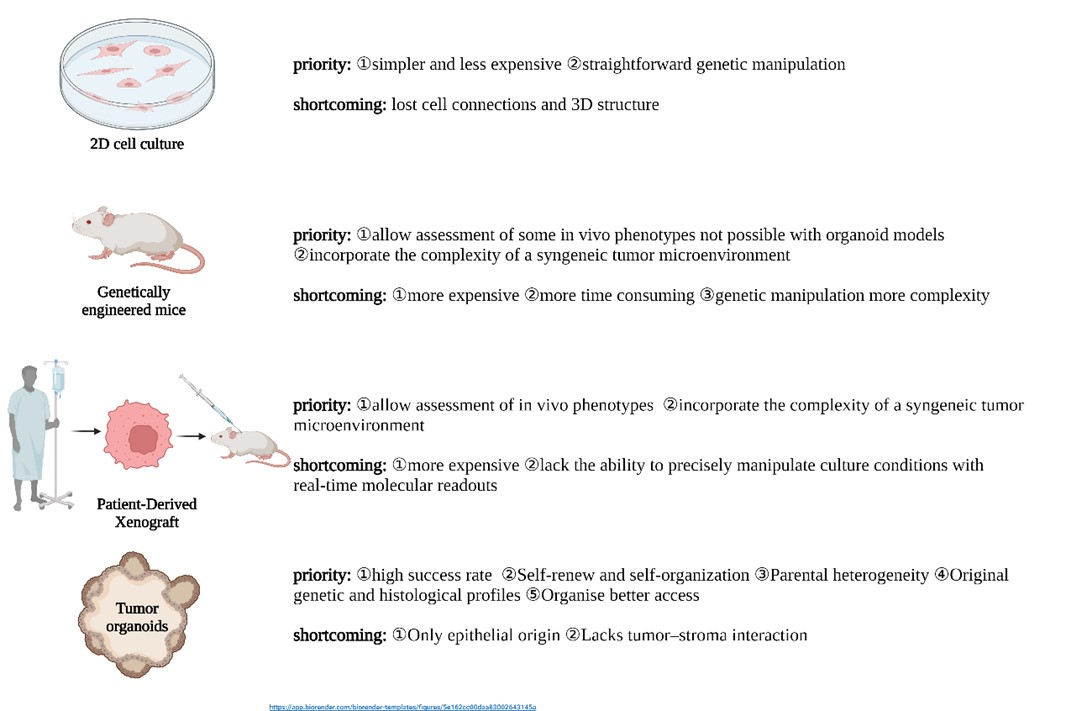

Supplement: Supplementary file 2 [file Image_2.jpeg]
